# Successful Targeting and Disruption of an Integrated Reporter Lentivirus Using the Engineered Homing Endonuclease Y2 I-*Ani*I

**DOI:** 10.1371/journal.pone.0016825

**Published:** 2011-02-09

**Authors:** Martine Aubert, Byoung Y. Ryu, Lindsey Banks, David J. Rawlings, Andrew M. Scharenberg, Keith R. Jerome

**Affiliations:** 1 Vaccine and Infectious Disease Division, Fred Hutchinson Cancer Research Center, Seattle, Washington, United States of America; 2 Department of Laboratory Medicine, University of Washington, Seattle, Washington, United States of America; 3 Department of Pediatrics, University of Washington and Seattle Children's Hospital, Seattle, Washington, United States of America; University of Liverpool, United Kingdom

## Abstract

Current antiviral therapy does not cure HIV-infected individuals because the virus establishes lifelong latent infection within long-lived memory T cells as integrated HIV proviral DNA. Here, we report a new therapeutic approach that aims to cure cells of latent HIV infection by rendering latent virus incapable of replication and pathogenesis via targeted cellular mutagenesis of essential viral genes. This is achieved by using a homing endonuclease to introduce DNA double-stranded breaks (dsb) within the integrated proviral DNA, which is followed by triggering of the cellular DNA damage response and error-prone repair. To evaluate this concept, we developed an *in vitro* culture model of viral latency, consisting of an integrated lentiviral vector with an easily evaluated reporter system to detect targeted mutagenesis events. Using this system, we demonstrate that homing endonucleases can efficiently and selectively target an integrated reporter lentivirus within the cellular genome, leading to mutation in the proviral DNA and loss of reporter gene expression. This new technology offers the possibility of selectively disabling integrated HIV provirus within latently infected cells.

## Introduction

The HIV epidemic continues to take an enormous toll in the lives of people throughout the world. While effective antivirals are widely available in developed countries, these drugs do not cure HIV, and require lifelong therapy. In the developing world, access to these drugs is severely limited. The single greatest advance for infected individuals would be the ability to cure their HIV infection.

Current treatments do not cure individuals of HIV because of the ability of HIV to establish lifelong latent infection within their hosts. For HIV, the major reservoir of latent infection consists of long-lived memory T cells containing integrated HIV proviral DNA. There have been suggestions of clearing this reservoir by inducing viral reactivation, presumably leading directly to death of latently infected cells or to their recognition and clearance by the immune system [1] [2]. To date, such approaches have not been successful. Even if all infected cells could be reactivated and eliminated, it is unclear what effects this would have on the immune function of the host - it is possible that systemic reactivation of HIV and widespread destruction of immune cells might lead to more severe clinical disease or even death. The ideal solution to the problem of HIV latency would be the ability to eliminate or otherwise disable integrated HIV without inducing death of infected cells, such that the latent virus is rendered incapable of further replication and pathogenesis.

Homing endonucleases (HEs) are enzymes that specifically target long (14–40 bp) DNA sequences and induce double-stranded breaks (dsb) [3]. Engineered HEs with altered DNA specificities are a promising tool for targeted gene therapy, since the specific locus of interest could be, in theory, targeted for cleavage and subsequently repaired by dsb-induced homologous recombination with a provided donor template [4], [5]. However, in mammalian cells dsb are predominantly repaired through non-homologous end-joining (NHEJ), a mechanism that does not require a homologous DNA template [6]. Importantly, NHEJ is mutagenic, since it joins broken ends with little or no homology, often resulting in small deletions and/or insertions surrounding the cleavage site [7]. This raises the possibility that an HE directed toward integrated proviral DNA might specifically induce mutagenesis of the viral sequences. Such mutagenesis could in turn prevent synthesis of virus-encoded proteins, preventing viral replication and continued pathogenesis. To evaluate this possibility, we constructed a reporter lentivirus containing the recognition sequence for the engineered homing endonuclease Y2 I-*Ani*I, and evaluated the effects of Y2 I-*Ani*I treatment on cells latently infected with the reporter lentivirus.

## Results

**Figure 1:**
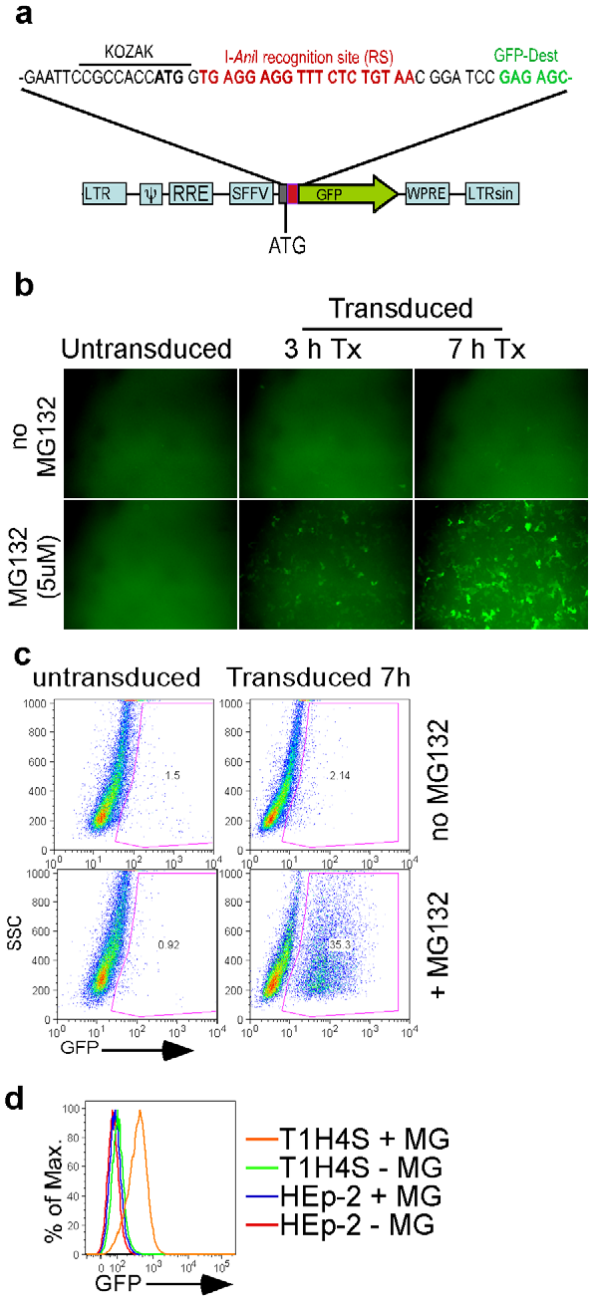
Cell line with integrated reporter lentivirus vector. (**a**) Schematic of the reporter lentivirus. (**b**) Immunofluorescence imaging of cells 3 days post transduction (dpt) with reporter lentivirus, with or without MG132 for 3 or 7 h. (**c**) Flow cytometry of the cells from panel b after 7 h treatment with MG132. (**d**) Flow cytometry of the clonal reporter (T1H4S) or parental (HEp-2) cell line after 5 h incubation with (+ MG) or without (−MG) 1 µM MG132.

To test if HEs can target and cleave integrated lentiviral genomes, we first developed a reporter construct with the recognition site for the engineered HE Y2 I-*Ani*I (which has the same cleavage specificity as its natural counterpart I-*Ani*I but has been redesigned for higher activity) [8] inserted between the translational start site and the gene encoding a short half-life GFP reporter (**Fig. 1a**). Upon cleavage of the recognition site by Y2 I-*Ani*I, imprecise repair via NHEJ should cause small deletions and/or insertions resulting in frame shifts, loss of the translational start site, or disruption of essential GFP coding sequences. Thus, successful HE attack can be monitored by loss of GFP expression. The expression cassette was then cloned into an integrating lentiviral vector under the SFFV promoter (**Fig. 1a**). The GFP reporter protein used in this study is destabilized by fusion with the PEST amino acid sequences from mouse ornithine decarboxylase (MODC), allowing rapid proteosomal degradation, enhanced protein turn over, and sensitive detection of changes in gene expression [9]. Therefore, GFP accumulation in HEp-2 cells transduced with the lentiviral reporter vector can only be detected following treatment with the proteosome inhibitor MG132 (**Fig. 1b and c**). A tissue culture model of integrated retroviral latency at low copy number was established by stably transducing cells with the integrating reporter lentivirus vector, and then cloning the cells using flow cytometry sorting and limiting dilution (**Fig. 1d**). The reporter cell lines showed similar growth properties as their parental lines and retained the ability to express GFP even after several months in culture.

**Figure 2 pone-0016825-g002:**
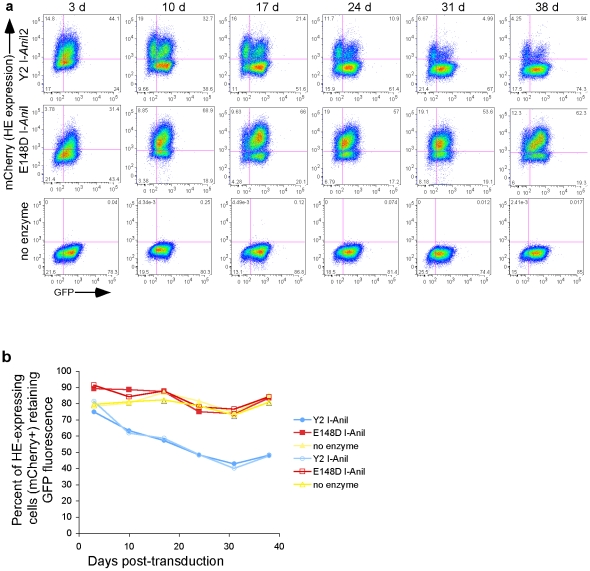
Targeting reporter gene in integrated lentivirus for mutagenesis by the homing endonuclease Y2 I-*Ani*I. (**a**) Reporter (GFP) fluorescence and Y2 I-*Ani*I expression (mCherry fluorescence) at the indicated days following transduction of the clonal reporter cell line T1H4S with lentiviral vector (moi of 2) expressing either the active enzyme Y2 I-*Ani*I or the control inactive enzyme E148D I-*Ani*I (as determined by mCherry expression), or control cells left untransduced (no enzyme, mCherry negative). This panel shows the data from one representative sample of a duplicate experiment. All cells were treated with 1 µM MG132 for 6 h prior to analysis. (**b**) Graphic representation of the data from the duplicate wells in the experiment depicted in Fig. 2a. Shown is the percent of the homing endonuclease-expressing cells (mCherry^+^ cell population) retaining reporter GFP fluorescence. The filled and open symbols correspond to the data from the duplicate wells. The percent of GFP^+^ cells was calculated as follows: {%GFP^+^ and mCherry^+^ cells x 100}/{Total % of mCherry^+^ cells}.

**Figure 3 pone-0016825-g003:**
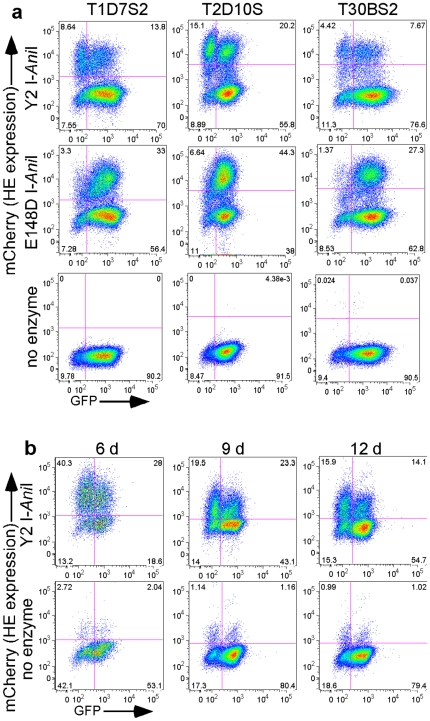
Targeting reporter gene in integrated lentivirus for mutagenesis using homing endonuclease in independent reporter cell lines. (**a**) Flow cytometry analysis of two different clonal (T1D7S and T2D10S) and the bulk uncloned (T30BS2) reporter cell lines transduced with lentiviral vector (moi = 2) expressing either active Y2 I-*Ani*I or inactive E148D I-*Ani*I enzyme, or left untransduced (no enzyme). (**b**) Flow cytometry analysis of GFP fluorescence and Y2 I-*Ani*I expression (mCherry fluorescence) at the indicated days following a second transduction of T1H4S cells with lentiviral vector expressing the active Y2 I-*Ani*I enzyme (moi = 2). All cells were treated with 1 µM MG132 for 6 h prior to analysis.

To determine whether the HE could access its target site embedded in the integrated lentiviral genome to generate dsb and DNA mutation, we transduced the reporter cells with a second lentiviral vector containing the gene for either Y2 I-*Ani*I or its inactive variant (E148D I-*Ani*I), linked via an IRES (Figs. 1–4) or T2A linker (Figs. 5–9) to the coding sequences of the mCherry fluorescent marker. GFP expression was then analyzed over time by flow cytometry. Over a 38-day period, cells expressing the active Y2 I-*Ani*I (clone T1H4S), but not the inactive enzyme, showed a progressive loss of reporter GFP expression (**Fig. 2a–b**). Similar results were obtained with two additional clonal reporter lines (T1D7S2 and T2D10S) as well as the bulk uncloned reporter cell line T30BS2 (**Fig. 3a**); all three cell lines showed a loss of GFP expression in the population of cells expressing the Y2 I-*Ani*I enzyme. Thus, the integrated viral sequences are accessible to Y2 I-*Ani*I attack, with no apparent effects due to the site of viral integration within the host genome. Cells were able to be transduced a second time with the Y2 I-*Ani*I-expressing lentivirus and showed good Y2 I-*Ani*I expression (**Fig. 3b**, top row), demonstrating that multiple rounds of homing endonuclease treatment are possible.

**Figure 4 pone-0016825-g004:**
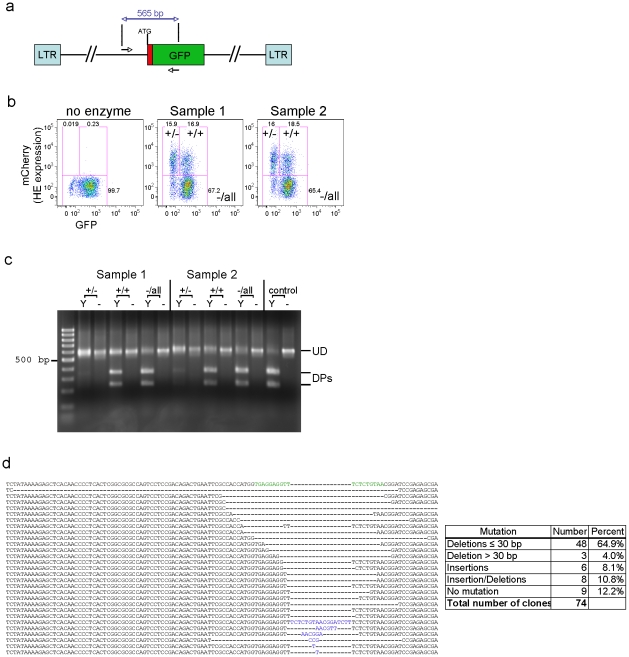
Mutagenesis of the reporter gene in integrated lentivirus following exposure to Y2 I-*Ani*I. (**a**) Location of PCR primers used to amplify the I-*Ani*I target site in reporter lentivirus integrated within cellular genomic DNA. (**b**) Three cell populations were sorted from T1H4S cells transduced with Y2 I-*Ani*I expressing lentiviral vector at 17 dpt: Y2 I-*Ani*I-expressing cells having retained (mCherry^+^; GFP^+^ or +/+) or lost (mCherry^+^; GFP^−^ or +/−) GFP expression, and cells negative for Y2 I-*Ani*I expression (mCherry^−^ or −/all). (**c**) PCR amplicons from the sorted cell populations shown in (b) were either exposed (Y) to Y2 I-*Ani*I enzyme *in vitro* or left unexposed (−). Full length undigested DNA (UD), digest products (DPs). The positive control consisted of PCR product directly amplified from reporter lentiviral vector DNA. (**d**) Sequence analysis of PCR amplicons from the sorted cell populations shown in (b). Left, representative mutated sequences. Top row shows wild type sequence with the I-*Ani*I target site indicated in green. Nucleotide insertions are indicated in blue. Right, summary of sequences analyzed. (GenBank accession numbers: HQ416600 to HQ416674).

**Figure 5 pone-0016825-g005:**
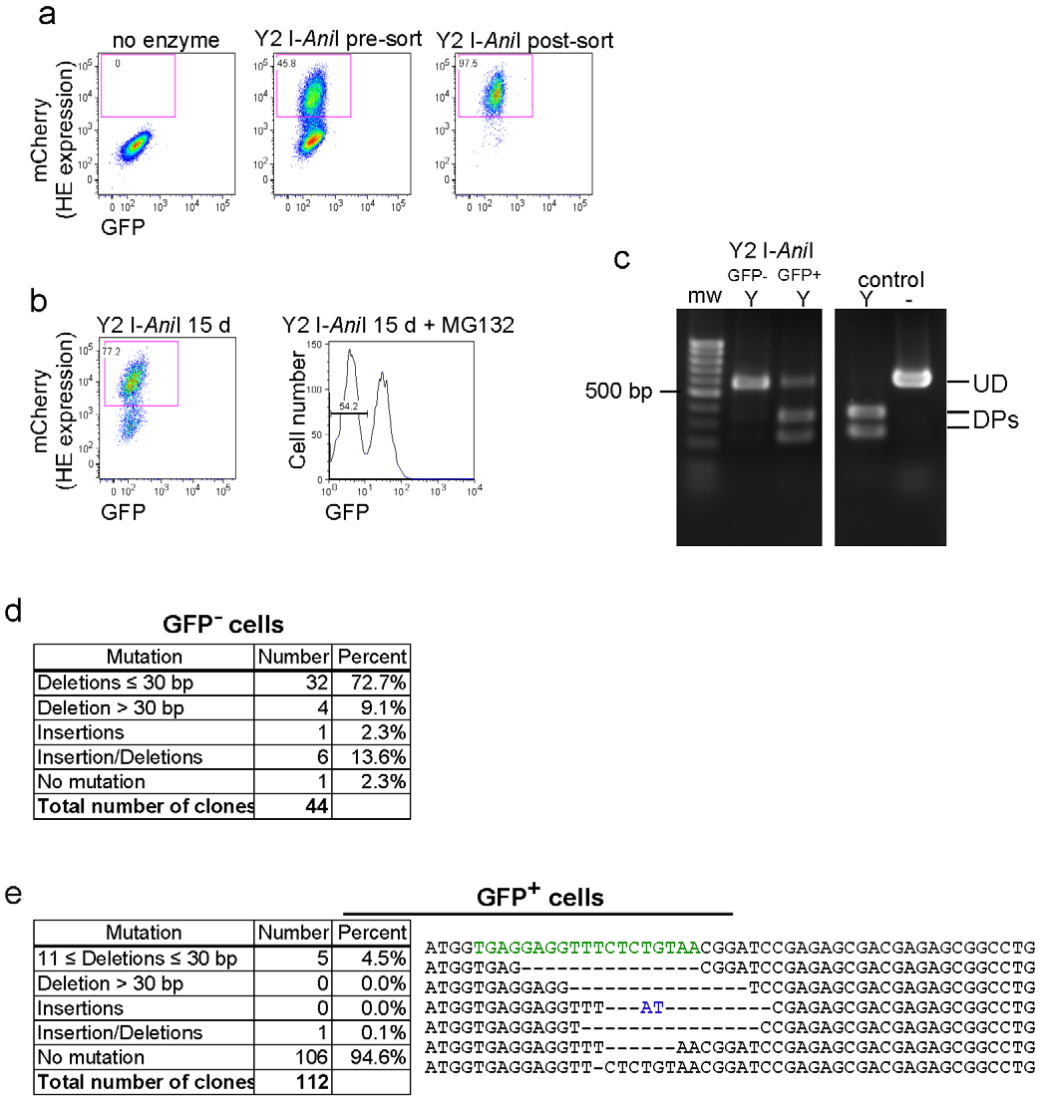
Analysis of the residual GFP^+^ cells after exposure to Y2 I-*Ani*I. (**a**) FACS of T1H4S cells transduced with Y2 I-*Ani*I expressing lentiviral vector at 3 dpt, used in panels (panel b-e). (**b**) Flow cytometry and FACS analysis of the cells from (panel a) and maintained in culture up to 15 dpt. Two populations were sorted based on GFP fluorescence. (**c**) Y2 I-*Ani*I digestion profiles of the PCR amplicons from cells in (panel b). Amplicons were either unexposed to (-) or exposed to (Y) Y2 I-*Ani*I enzyme. Full length undigested DNA (UD), digest products (DPs), control: PCR product from reporter lentiviral vector DNA. (**d**) Summary of sequence analysis of the target region in GFP- cells (GenBank accession numbers: HQ416556 to HQ416599). (**e**). Sequence analysis of the target region in GFP^+^ cells (e, left) and DNA mutations detected in the GFP+ population (e, right). Top row corresponds to the wild type sequence with the I-*Ani*I target site indicated in green. Nucleotide insertions are indicated in blue. (GenBank accession numbers: HQ416445 to HQ416555).

**Figure 6 pone-0016825-g006:**
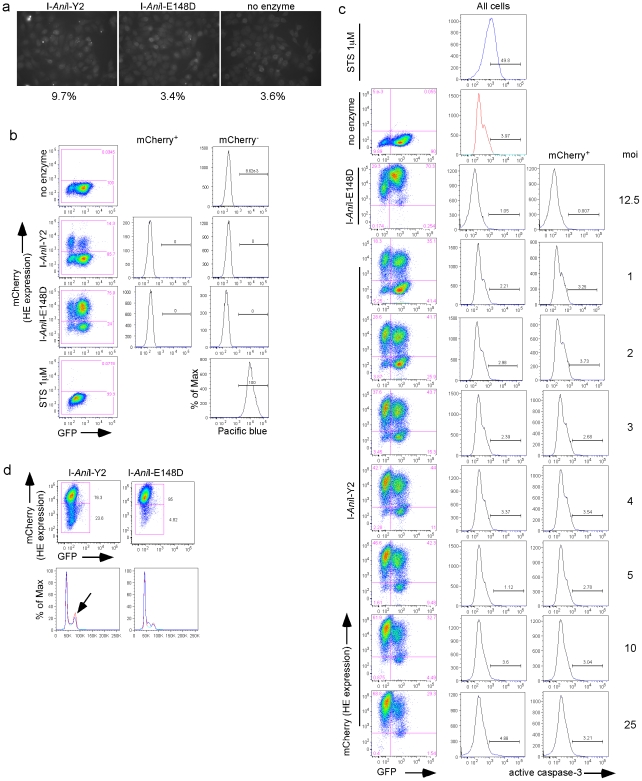
Lack of homing endonuclease toxicity. (**a**) Immunofluorescence images of cells stained for γ-H2AX. Nuclei were counterstained with DAPI. At least 330 cells were scored for the presence of foci under a 40X objective. (**b**) Live/dead analysis of cells 35 dpt. Left column, flow cytometric analysis for reporter GFP fluorescence and HE expression (mCherry fluorescence). The histograms from the middle (mCherry^+^ cells only) and right (mCherry^−^ cells only) columns show the results of the live/dead analysis; dead cells with compromised membranes stain with the amine-reactive fluorescent dye (Pacific blue). Positive control cells treated overnight with 1 µM staurosporine (STS, a cell death inducer) are shown in the bottom row. (**c**) Flow cytometry analysis for activated caspase-3. T1H4S cells were transduced at the indicated moi with lentiviral vector expressing either active Y2 I-*Ani*I or inactive E148D I-*Ani*I enzyme. At 3 dpt, cells were split into duplicate wells and assayed for cell death. One well for each condition was used for flow cytometric analysis of reporter GFP fluorescence and HE expression (mCherry fluorescence) after 6 h treatment with MG132. The second well of cells was used to assay for apoptosis by caspase-3 staining as described in Material & Methods. The positive control for apoptosis was obtained by treating T1H4S cells left unexposed to HE-expressing lentivirus with 1 µM staurosporine STS overnight. The left column shows the GFP and mCherry fluorescence analysis, and the middle (total cell population) and right (mCherry^+^ cells only) columns present the results of the caspase-3 analysis for each condition. (**d**) Cell cycle analysis of T1H4S cells transduced with lentiviral vector expressing either active Y2 I-*Ani*I or inactive E148D I-*Ani*I enzyme for 6 days, sorted for mCherry expression, and kept for 22 more days in culture. Cells were not treated with MG132 prior to staining and flow cytometry analysis. Blue, mCherry^−^ cells; red mCherry^+^. Arrow indicates the increased delay in G2 among cells exposed to Y2 I-*Ani*I.

**Figure 7 pone-0016825-g007:**
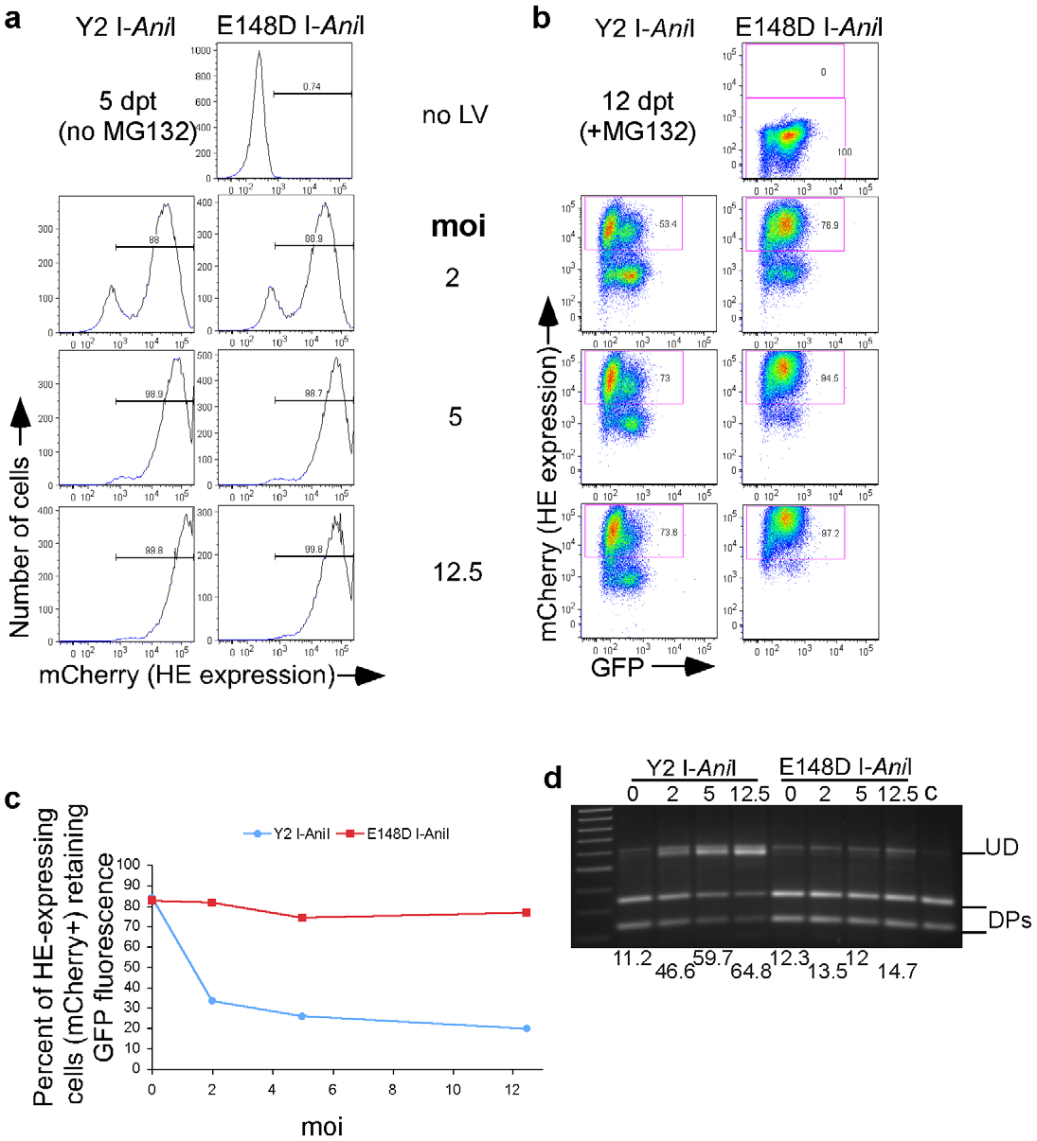
Mutagenesis of the reporter gene in integrated lentivirus following exposure to increasing amount of Y2 I-*Ani*I. (**a**) Y2 I-*Ani*I expression (mCherry fluorescence) at 5 dpt **and** (**b**) reporter GFP fluorescence and Y2 I-*Ani*I expression (mCherry fluorescence) at 12 dpt of the reporter cell line T1H4S transduced with the indicated moi of lentiviral vector expressing either active Y2 I-*Ani*I or inactive E148D I-*Ani*I enzyme. The experiments in **Fig. 2** **–** [Fig pone-0016825-g003] [Fig pone-0016825-g004] [Fig pone-0016825-g005] **6** used LV-Y2 I*-Ani*I and LV-E148D I-*Ani*I at a moi of 2. (**c**) Graphic representation of the percent of homing endonuclease-expressing cells (mCherry^+^ cell population) having retained GFP fluorescence. The percent of GFP^+^ cells was calculated as follows: {%GFP^+^ and mCherry^+^ cells x 100}/{Total % of mCherry^+^ cells}. (**d**)Y2 I-*Ani*I digestion profiles of the PCR amplicons containing the Y2 I-*Ani*I target region from the unsorted cell populations of panel (b). Full length undigested DNA (UD), digest products (DPs). Numbers below the figure represent percent of undigested full length DNA remaining after Y2 I-*Ani*I digestion.

**Figure 8 pone-0016825-g008:**
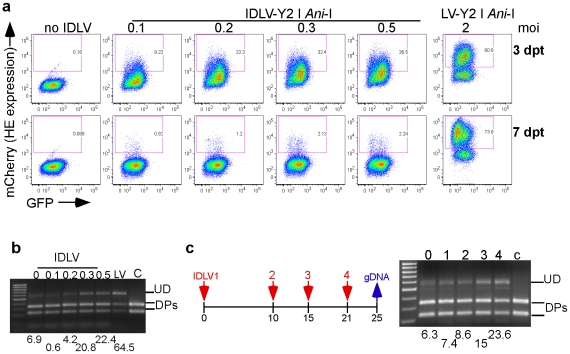
Mutagenesis of the reporter gene in integrated lentivirus following transient exposure to Y2 I-*Ani*I. (**a**) Reporter GFP fluorescence and Y2 I-*Ani*I expression (mCherry fluorescence) at 3 and 7 dpt of the reporter cell line T1H4S transduced with the indicated moi of non integrating (IDLV) or integrating (LV) lentiviral vector expressing active Y2 I-*Ani*I enzyme, (**b**) Y2 I-*Ani*I digestion profiles of PCR amplicons containing the Y2 I-*Ani*I target region obtained with the 7 dpt unsorted cells shown in panel (a). Numbers below the figure represent percent of undigested full length DNA remaining after Y2 I-*Ani*I digestion. In (b) and (c), full length undigested DNA (UD), digest products (DPs); the positive control (“C”) consisted of PCR product directly amplified from reporter lentiviral vector DNA. (**c**) Left, transduction schedule of the reporter cell line T1H4S with non integrating (IDLV) Y2 I-*Ani*I expressing lentiviral vector at an moi of 0.5. Red arrows indicate the transduction time points and the blue arrow the time of gDNA isolation from the unsorted cell populations. Right, Y2 I-*Ani*I digestion profiles of the PCR amplicons containing the Y2 I-*Ani*I target region from the unsorted cell populations at day 25. Numbers below the figure represent percent of undigested full length DNA remaining after Y2 I-*Ani*I digestion.

**Figure 9:**
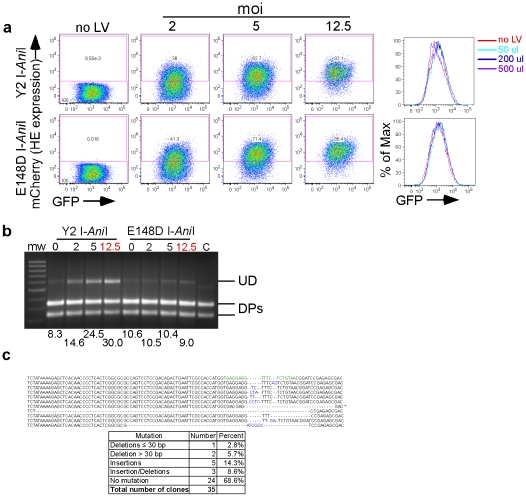
Mutagenesis of the integrated lentivirus reporter gene in T cells following exposure to Y2 I-*Ani*I. (**a**) Reporter GFP fluorescence and Y2 I-*Ani*I expression (mCherry fluorescence) at 13 dpt of a Jurkat T reporter cell line transduced with the indicated moi of lentiviral vector expressing either active Y2 I-*Ani*I or inactive E148D I-*Ani*I enzyme. Right panels show the overlaid GFP fluorescence histograms of the cells expressing the enzyme (mCherry^+^ cells from the dot plots on the left side). The no LV histograms correspond to the mCherry negative population. (**b**) Y2 I-*Ani*I digestion profiles of the PCR amplicons containing the Y2 I-*Ani*I target region obtained from the unsorted cells from panel (a). Full length undigested DNA (UD), digest products (DPs). The positive control (“C”) consisted of PCR product directly amplified from reporter lentivirus. (**c**) Sequence analysis of PCR amplicons from cells transduced at an moi of 12.5 from panel (a–b). Nucleotide insertions are indicated in blue. (GenBank accession numbers: HQ432772 to HQ432804).

In order to establish the genetic basis for the loss of GFP expression observed in Y2 I-*Ani*I expressing cells, PCR amplification of the region containing the target site was performed using *Pfx* and primers located on either side of the Y2 I-*Ani*I recognition sequence **(** **Fig. 4a**). Amplification was done on genomic DNA obtained at 17 d post-transduction (dpt) from three sorted cell populations (**Fig. 4b**): Y2 I-*Ani*I-expressing cells having retained (mCherry^+^/GFP^+^ or +/+) or lost (mCherry^+^/GFP^−^ or +/−) GFP expression, and all cells negative for Y2 I-*Ani*I expression (mCherry^−^/all or −/all). First, mutation of the target site was demonstrated by *in vitro* digestion of the PCR amplicon with recombinant Y2 I-*Ani*I enzyme. As seen in **Fig. 4c**, the amplicon from cells having lost GFP expression (+/−) was almost totally resistant to Y2 I-*Ani*I digestion, while the majority of target site DNA from cells retaining GFP expression (+/+) or negative for Y2 I-*Ani*I expression (−/all) could be cleaved by Y2 I-*Ani*I. Sequencing of the region containing Y2 I-*Ani*I target site from cells having lost GFP expression revealed a series of genetic mutations, the majority being small deletions (64.9%≤30 bp), with a lesser number of larger deletions (4%, some over 200 bp); insertions (8.1%) or a combination of insertion/deletions (10.8%) (**Fig. 4d**, GenBank accession numbers: HQ416600 to HQ416674). These data confirmed that the loss of GFP expression and the resistance to Y2 I-*Ani*I digest observed is indeed due specifically to HE-induced mutation of the integrated proviral Y2 I-*Ani*I target region.

After exposure to Y2 I-*Ani*I, a proportion of cells remained positive for reporter GFP expression (**Fig. 2a**). A possible explanation is that these cells might have undergone genetic mutations destroying the Y2 I-*Ani*I recognition site but preserving protein expression (for example, small deletions of 3 or 6 bp), and thus be refractory to subsequent Y2 I-*Ani*I attack. To evaluate this possibility, a new set of experiments was done and sequencing analysis of the target site compared between cells having retained or lost GFP expression following Y2 I-*Ani*I exposure. In this set of experiments, reporter cells were transduced with Y2 I-*Ani*I expressing lentivirus, sorted for Y2 I-*Ani*I expression 3 dpt (**Fig. 5a**), passaged in culture and then sorted based on GFP expression at 15 dpt (**Fig. 5b**). Sequence analysis of the target site region for the cells having lost GFP expression yielded similar results as described above, showing resistance to Y2 I-*Ani*I digestion *in vitro* (**Fig. 5c**) and mutation of Y2 I-*Ani*I target region (**Fig. 5d**, GenBank accession numbers: HQ416556 to HQ416599). In contrast, analysis of the Y2 I-*Ani*I target region from residual GFP+ cells revealed that the majority of the DNA remained sensitive to Y2 I-*Ani*I digestion *in vitro* (**Fig. 5c**) suggesting that the target site remained intact. Sequence analysis showed that although a few clones presented the predicted short deletions in multiples of 3 bp (**Fig. 5e**, GenBank accession numbers: HQ416445 to HQ416555), the majority (95%) retained the wild-type recognition sequence, and thus would be expected to be susceptible to ongoing attack by Y2-*Ani*I.

A major concern regarding the clinical use of HEs is the possibility of toxic effects. Over an extended period in culture, the proportion of cells expressing Y2 I-*Ani*I (mCherry+) decreased (**Fig. 2a**). This was not observed in cells expressing the inactive variant E148D I-*Ani*I (**Fig. 2a**), excluding the possibility that loss of mCherry expression was predominantly due to gene silencing. DNA dsb in the mammalian genome triggers DNA damage response pathways, which lead to cell cycle arrest, activation of repair mechanisms, and, if damage is too extensive to repair, initiation of cell death [10], [11]. An early step in recruiting and localizing DNA repair proteins is the phosphorylation of the histone H2AX (γ-H2AX) and localization of this protein into discreet foci. These foci represent DNA dsb in a 1∶1 manner and can be used as a biomarker for DNA damage [12]. Therefore, we investigated if Y2 I-*Ani*I nuclease induced extensive DNA damage (dsb), as might be observed if there were substantial cleavage of off-target sequences. As expected, foci were more frequently observed in cells expressing the active Y2 I-*Ani*I than in cells expressing the inactive variant or non-transduced cells (no enzyme) (**Fig. 6a**). Multiple bright foci were rarely observed in a single cell; most positive cells had a single bright focus. No detectable cell death was observed in Y2 I-*Ani*I exposed cells, even those exposed to high levels of the enzyme (**Fig. 6b–c**), suggesting that toxicity of the HE could not explain the decrease in Y2 I-*Ani*I-expressing cells over time. Instead, the Y2 I-*Ani*I expressing cells showed an increased proportion of cells in G2 phase compared to cells not expressing Y2 I-*Ani*I or cells expressing the inactive enzyme (**Fig. 6d**). Thus, it appears that the cell cycle delay required for repair of HE-induced dsb puts HE-expressing cells at a slight but reproducible growth disadvantage in mixed cultures, compared to cells not expressing HEs.

In the experiment described in **Fig. 2**, a loss of GFP expression was observed in 50% of Y2 I-*Ani*I-expressing cells by 24 days after introduction of the HE. By this time, the proportion of cells still expressing Y2 I-*Ani*I had significantly decreased. To determine if more complete disruption of integrated lentiviral sequences could be obtained in a shorter period of time, the reporter cell line was exposed to increasing amounts of Y2 I-*Ani*I-expressing lentivirus. At 5 dpt with higher amounts of lentivirus, nearly 100% of the cells expressed Y2 I-*Ani*I (**Fig. 7a**), and more than 70% were still expressing at 12 dpt (**Fig. 7b**). By 12 dpt with the higher transduction levels, 75-80% of Y2 I-*Ani*I-expressing cells showed loss of GFP fluorescence (**Fig. 7b**, left panels, and **Fig. 7c**). When the target site DNA in those cell populations was analyzed for susceptibility to *in vitro* Y2 I-*Ani*I digestion, more complete protection from cleavage was seen (**Fig. 7d**) compared to the conditions (moi = 2) used in the previous experiments (**Fig. 4c**).

In all the experiments described above, integrating lentivirus vectors were used to introduce I-*Ani*I nuclease into the target cells, assuring high level and sustained expression of the enzyme. However, integrating lentivirus vectors are unlikely to be acceptable for clinical use. Therefore, we evaluated the use of a non-integrating lentivirus vector (integration-deficient lentivirus, IDLV) to transiently express Y2 I-*Ani*I nuclease. After transduction of the target reporter line with the IDLV expressing Y2 I-*Ani*I, flow cytometric as well as I-*Ani*I target site DNA analysis were performed to detect mutation of the target site. After a one time exposure to IDLV-Y2 I *Ani-*I, cells became transiently positive (mcherry+) for Y2 I-*Ani*I expression (**Fig. 8a**, compare top and bottom rows) while cells exposed to integrating lentivirus (LV) retained Y2 I-*Ani*I expression. The analysis of the Y2 I-*Ani*I digestion susceptibility of the target site showed that while the expression of Y2 I-*Ani*I was transient, it was sufficient to lead to the accumulation of target site sequences resistant to Y2 I-*Ani*I digestion (**Fig. 8b**). Since the proportion of resistant target sites was relatively low, cells were repeatedly exposed to IDLV-Y2 I-*Ani*I to determine if the proportion of mutated sequences could be increased. The cells exposed to IDLV-Y2 I-*Ani*I (cells transduced with an moi of 0.5; **Fig. 8a**,) were either maintained in culture or exposed 1 (IDLV2), 2 (IDLV3) or 3 (IDLV4) additional times to IDLV-Y2 I-*Ani*I (moi of 0.5; **Fig. 8c**) before PCR amplification of the target site region and Y2 I-*Ani*I DNA digestion analysis. The percentage of DNA target site resistant to Y2 I-*Ani*I digestion detected increased with the number of exposures to Y2 I-*Ani*I (**Fig. 8c**). This suggests that repeated exposure to transient expression of Y2 I-*Ani*I is capable of achieving higher percent target site mutagenesis.

Finally, we asked whether similar results could be obtained in latently infected T cells, since in humans, HIV establishes lifelong latent infection within long-lived memory T cells [13]. We therefore established a Jurkat T cell line stably transduced with the reporter system described above. The Jurkat T reporter cells were exposed to integrating lentiviral vector expressing either the active or inactive form of Y2 I-*Ani*I. Thirteen dpt, the cells were analyzed by flow cytometry for GFP expression and Y2 I-*Ani*I target site DNA analysis performed on the unsorted population exposed to the highest amount of lentivirus. Although GFP expression in the Jurkat cells was only slightly above background, a reduction in GFP fluorescence was observed in cells exposed to the largest amount of lentivirus expressing Y2 I-*Ani*I (**Fig. 9a**). To confirm the ability of Y2 I-*Ani*I to successfully target provirus in T cells, we PCR amplified the region containing the target site. The resulting amplicon was resistant to *in vitro* Y2 I-*Ani*I digestion (**Fig. 9b**), suggesting mutation of the site. Sequencing of the amplicon demonstrated a variety of insertions and or deletions (**Fig. 9c**, GenBank accession numbers: HQ432772 to HQ432804),confirming the function of HEs in T cells.

## Discussion

HIV establishes lifelong latency within memory T cells, and thus current therapies can not eradicate the infection, even if they successfully inhibit viral replication. The only hope for cure of HIV infection lies in complete elimination of latently infected cells or the integrated provirus they contain. Currently, there is no way to selectively target and eliminate latently infected T cells. Some studies have attempted to purge the T cell compartment by non-specifically activating HIV within memory T cells, in hopes that triggering viral replication would result in the destruction of latently infected cells [1], [2]. Such approaches have to date proven unsuccessful. Even if it were possible to target and destroy latently infected T cells with some degree of specificity, it is unclear what effects the widespread T-cell destruction would have on the ability of the infected individual to reconstitute an effective immune response.

Because of the difficulties and potential dangers inherent in targeting the latently infected cells themselves, an attractive approach would be to selectively attack the integrated provirus within these cells. Until recently this has been a largely hypothetical possibility, but recent advances in the use of DNA modifying enzymes have brought this closer to reality. In one approach, the Buchholz and colleagues [14] modified the Cre recombinase to recognize sequences within the LTR of HIV strain TZB0003. Exposure of latently infected cells to the modified recombinase led to excision of provirus and the effective cure of the cells. Unfortunately, the LTR sequence of strain TZB0003 is atypical for HIV, and was chosen for these studies because of its similarity to the DNA sequence recognized by the wild type Cre recombinase. The corresponding LTR sequences from other HIV strains are much more divergent from the wild type Cre recognition sequence, and thus it is unlikely that this approach can ever be generalized to clinically relevant HIV strains.

Although the excision of integrated provirus is conceptually attractive, we would argue that excision of provirus is unnecessary for the effective cure of latent HIV infection. It is well known that the human genome is comprised in large part of the remnants of ancient retroviral infections; yet these remnants do not cause disease. The only prerequisite for an effective genetic cure of HIV infection is that the integrated provirus be mutated sufficiently to prevent production of progeny virus and continued pathogenesis. It is in this context that we evaluated the possibility of using an engineered homing endonuclease to specifically target integrated provirus, leading to NHEJ and the induction of mutation.

Recently, a number of studies have focused on strategies to reengineer HEs to recognize DNA sequences of interest, mainly for applications in gene therapy [15], [16], [17], [18], [19], [20], [21], [22], [23]. These applications rely on repair of the HE-induced dsb via homologous recombination with a supplied template with the desired (usually wild-type) sequence. Unfortunately for such studies, in mammalian cells dsb are often repaired via NHEJ, a process prone to DNA deletions and other mutations. Here, we have utilized the existing HE Y2 I-*Ani*I to demonstrate that HEs directed toward integrated proviral sequences can induce dsb and mutation of integrated proviral DNA. Similar results could presumably be obtained using other DNA double-strand cleaving enzymes such as the zinc-finger nucleases (ZFNs). Indeed, ZFNs have recently been used to mutate CCR5, which serves as a co-receptor for certain strains of HIV [24], and ZFNs have also been designed that target hepatitis B virus [25]. While ZFNs are more easily redirected toward desired sequences than HEs, the ZFNs may also be less specific, raising concerns regarding off-target cleavage and mutation of undesired genomic sites. We believe that the highly specific HEs are likely to provide a more viable pathway to ultimate clinical use, although formidable challenges remain in retargeting them toward HIV sequences. Among these challenges are the rapid mutation of HIV and the resulting diversity of HIV sequences; these will likely require the use of multiple HIV-specific HEs, each recognizing well-conserved targets. Targeting HEs to the relevant long-lived cells comprising the HIV reservoir is also a major issue, and will require significant improvements upon our existing delivery systems. Nevertheless, our data demonstrate that engineered HEs can specifically and efficiently target specific sequences embedded into an integrated reporter lentivirus, resulting in mutation and the inability to synthesize lentiviral reporter-encoded protein. The results suggest that reengineering HEs to target viral DNA sequences could be a viable strategy to mutate integrated HIV provirus, thereby eliminating viral replication and pathogenesis, effectively curing latently infected cells.

## Materials and Methods

### Cell lines

Jurkat and HEp -2 cells were obtained from ATCC and passaged in RPMI supplemented with 10% fetal bovine serum (FBS), Sodium Pyruvate, glutamate and DMEM supplemented with 10% FBS, respectively.

### Lentiviral vector

pAniI-RS-TurboGFP was generated from pTurbo GFP (Evrogen) by removal of the start codon of GFP using the following primers 5′-CGGGATCCGAGAGCGACGAGAGCGGCCTGC-3′ and 5′-TGCTCCACGGTGGCGTTGCTGCGGATGATC-3′ and cloning *Bam*HI-digested amplicon back into the *Bam*HI-*Xmn*I digested pTurbo GFP, followed by cloning of the I-*Ani*I target sequence, using the primers 5′-ATTCGCCACCATGGTGAGGAGGTTTCTCTGTAACG-3′ and GATCCGTTACAGAGAAACCTCCTCACCATGGTGGCG-3′, into the *Bam*HI-*Eco*RI-digested pTurbo GFP. A second start codon in the N-terminal sequence of the GFP gene was also removed.

A lentiviral reporter vector (pRRL.SFFV.I-*Ani*I-RS-GFP) was constructed by subcloning the *Eco*RI (blunted)-*Xba*I fragment of the plasmid pI-*Ani*I-RS-TurboGFP containing the I-*Ani*I reporter site into klenow-treated *Tth*111I and *Spe*I sites of the pRRL.SFFV vector derived from pRRLPGK-GFP[26]. pCVL.SFFV.Y2 I-*Ani*I.IRES.mCherry and pCVL.SFFV.E148D I-*Ani*I.IRES.mCherry were used to transduce cells in the experiments presented in **Fig. 1** **–** [Fig pone-0016825-g002] [Fig pone-0016825-g003] **4**. pCVL.SFFV.Y2 I-*Ani*I.2A.mCherry and pCVL.SFFV.E148D I-*Ani*I.2A.mCherry were the integrating lentiviral vectors used for the transduction experiments presented in **Fig. 5** **–** [Fig pone-0016825-g006] [Fig pone-0016825-g007] [Fig pone-0016825-g008] **9**. For transient expression of Y2-I-*Ani*I, a lentiviral vector with a short form of EF1α driving Y2 I-*Ani*I.2A.mCherry was constructed and used to produce integration deficient lentiviral vector (IDLV). VSV-G-pseudotyped lentiviral vectors were produced by transient transfection of 293T cells as described elsewhere [27]. Lentiviral stocks were titrated by transducing 2×10^5^ cells with 2-fold increased volume of lentiviral supernatant and determination of the number of transduced cells by flow cytometry analysis for the fluorescent marker 3–4 days post-transduction. The titer (ifu/ml) was calculated as {(number of transduced cells x % fluorescent positive cells)/100}/volume of viral supernatant used in ml.

### Cell transduction

For HEp-2, 2×10^5^ cells per well were placed in one well of a12-well plate and either left to adhere for 12–16 h before being transduced with the lentivirus construct indicated, in 1 ml culture medium containing 4 µg/ml polybrene. The next day, the transduction medium was replaced with fresh culture medium and incubated for 3–4 more days before GFP and/or mCherry fluorescence analysis. Jurkat cells (2×10^5^ cells per well) were transduced with the lentivirus vectors in 1 ml culture medium containing 4 µg/ml polybrene, incubated at 37°C for 6 h minimum in the transduction medium, 0.5–1 ml fresh medium added and further incubated 3–4 days before analysis.

### Construction of reporter target cell line

After transduction of the cells with the lentiviral reporter pRRL.SFFV.AniI-RS-GFP, clones were isolated by limiting dilution and fluorescence-activated cell sorting (FACS). Clones were cultivated and assessed for GFP expression. The bulk cell line was established by flow cytometric sorting of the GFP positive cell population after transduction. No decrease of GFP expression was observed after several months in culture (>2 months).

### DNA analysis

Genomic DNA was isolated using the DNeasy Blood & Tissue Kit (Qiagen) from either sorted cell populations or transduced cells collected by trypsinization or centrifugation for adherent or non-adherent cells, respectively. PCR amplification of the DNA sequence containing the I-*Ani*I target site was performed as follows. Fifty ng of genomic DNA was used to amplify a 556 bp DNA sequence containing the Y2 I-*Ani*I recognition sequence from the integrated pRRL.SFFV.AniI-RS-GFP lentivirus, using Platinum *Pfx* DNA polymerase (Invitrogen), primers: 5′-GGAAGCTTGCCAAACAGGATATCTGCGGTGAGC-3′ and 5′-GACTAGTCGGGTAGGTGCCGAAGTGGTAGAAGC-3′, and the following program: 94°C 2 min., for 35 cycles 94°C 15 sec, 60°C 15 sec, 68°C 30 sec, then ended with 68°C 10 min.

For sequencing analysis, amplicons were subsequently purified after electrophoresis on a 1% agarose gel, using QIAquick gel extraction kit or cleaned up using QIAquick PCR purification kit (Qiagen) per the manufacturer's recommendations. The purified PCR amplicons were cloned into PCR®4Blunt-TOPO® vector using Zero Blunt®TOPO® PCR Cloning kit for sequencing (Invitrogen) as per manufacturer's recommendations. Briefly, 5 µl of purified PCR product were ligated into the vector during a 20 min incubation at room temperature, and transformed into One Shot® TOP10 competent cells. Transformants were selected by plating onto LB-agar plate containing 50 µg/ml Kanamycin. Colonies were grown overnight in LB containing 50 µg/ml Kanamycin. Plasmid DNA was isolated using the PureLink Quick plasmid miniprep kit (Invitrogen) as per manufacturer's protocol. The presence of an insert was confirmed by *Eco*RI digest of the plasmid DNA, followed by electrophoresis on 1% agarose gel. The plasmid DNAs were also subjected to *in vitro* Y2 I-*Ani*I digest using purified Y2 I-*Ani*I enzyme as described by Takeuchi et al [8], followed by electrophoresis onto 2% agarose gel. After staining the DNA with ethidium bromide solution, gel images were captured using an AlphaImagerTM 3400 (AlphInnotech) system and the intensity (I) of the DNA fragments determined using AlphaEaseFC software tools. The percent of undigested full length DNA was calculated as follows: [I _full length undigested DNA_/(I_full length undigested DNA_+I_digested products_)]x100. The insert of each isolated plasmid was sequenced using 5 µl of plasmid DNA, 5 pmol primer T3 (sequence) or M13F (sequence) and BigDye sequencing reaction (FHCRC, Shared resources) using the program 96°C 1 min., for 25 cycles 96°C 10 sec, 50°C 5 sec, 60°C 4 min.

### Flow cytometry

To analyze GFP fluorescence in the HEp-2 reporter cell lines, cells were treated with 1 µM MG132 (Calbiochem) for 6 h or otherwise indicated, and kept overnight at 4°C in 1% paraformaldehyde in PBS prior to analysis on a BD LSRII flow cytometer. GFP fluorescence analysis in the reporter Jurkat cell line was performed on a BD LSRII flow cytometer immediately after 6 h incubation with 1 µM MG132. For cell death analysis, cells were collected by trypsinization, washed in PBS, and then assayed for cell viability using Live/Dead cell stain kit (Invitrogen, as per the manufacturer's recommendations) which stains dead cells with compromised membranes after reaction with the amine-reactive fluorescent dye (Pacific blue). Apoptosis was also evaluated by staining for active-caspase-3 as previously described [28] with the following modifications. After fixation with paraformaldehyde, cells were permeabilized with 0.1% Triton X-100 for 10 min and stained with FITC conjugated anti-active caspase-3 antibody diluted in 50 µl 0.1% Triton X-100 1% BSA solution at room temperature.

Cell sorting for mCherry fluorescence was performed on a BD FACSAria II and for GFP fluorescence on a BD FACSVantage (FHCRC, shared resources).

All the data were analyzed using FlowJo software (TreeStar).

### γ-H2AX immmunofluorescence

Cells (0.5–1×10^6^) were grown overnight on glass coverslips prior to staining γ-H2AX as described previously [29]. Briefly, the cells were fixed with 2% paraformaldehyde in PBS for 20 min, permeabilized with 0.5% Triton X-100 in PBS for 10 min, and incubated in blocking buffer (PBS +3% bovine serum albumin +0.1% Tween 20) for 30 min. Anti-γ-H2AX (JBW301, 1∶1,000; Upstate) and FITC conjugated anti-mouse (1∶1000: Jackson ImmunoResearch) were used and nuclei were counterstained with 4′,6-diamidino-2-phenylindole (1 µg/mL). Coverslips were mounted on slides in Vectashield (Vector Laboratories). Images were acquired using a microscope (TE2000, Nikon) equipped with a 40x immersion objective (1.3 numerical aperture) and a CCD camera (CoolSNAP ES, Photometrics) and analyzed using MetaVue (Universal Imaging). At least 330 cells were scored for the presence of foci.
